# Supplementary material for: Using a Modified Delphi Approach and Nominal Group Technique for Organisational Priority Setting of Evidence-Based Interventions That Advance Women in Healthcare Leadership
Source: Int J Environ Res Public Health. 2022 Nov 17;19(22):15202. doi: 10.3390/ijerph192215202 (PMC9690121; doi:10.3390/ijerph192215202)
Supplement: Supplementary file 1 [file ijerph-19-15202-s001.zip › ijerph-1989743-supplementary.pdf]

Supplementary File S1. Organisational Priority Setting Framework

Criteria 1: Potential of proposal
Is there a strong rationale/evidence base for the proposed initiative to improve outcomes for women? Is there evidence for the proposed approach to addresses a gap in current organisational practice at the organisation? Is it feasible to institute? (e.g. Is it proven and likely to improve outcomes for women in leadership?

Criteria 2: Prevention
Is there potential to prevent significant problems or secondary impacts for women in the workforce? (e.g. Can these priorities prevent poor career outcomes for women)? Can they alleviate the ongoing and disproportionate impacts of the COVID-19 pandemic on women?

Criteria 3: Position
Consider geographical/ regional issues and location of services/ expertise. What are the inequities that can be improved by this initiative? Is there a need to adapt for regional locations? (e.g. Would these initiatives improve outcomes for women in both metro and regional/rural area, would it impact vulnerable groups of women)?

Criteria 4: Participation
Is engagement/ collaboration critical to success? Are there clear drivers for stakeholders to engage and collaborate? Are there existing relationships between stakeholders that can be leveraged to support these initiatives? Is there potential for strong engagement in the proposed initiatives? (e.g. is there a role/ fit for this initiative with the Epworth sites)?

Criteria 5: Policy
Does the problem or the potential solution align with current policy directions, including strategic priorities? (e.g. Do gender equity initiatives align with policy priorities)?

Criteria 6: Proposed Strategy
Does the proposal align with the purpose of the 2025 strategic areas? (e.g. Will these gender-based initiatives align with our purpose to grow, develop and succeed our workforce? Do they increase competition for talent? set a new benchmark for patient care? and work with our doctors and key strategic partners to deliver care across Victoria in new and innovative ways?

Criteria 7: Proposed Transformation
Will addressing this problem/ taking this approach collaboratively, lead to a transformational change for women in healthcare and subsequently improve health outcomes? Does this transformation consider projected market shifts in the private healthcare landscape and reflect changing consumer expectations? (e.g. Would a gender-based initiative transform the workforce and impact on care and outcomes?

Note***Successful delivery of strategic needs:

- Active and visible leadership: aligned leaders that are focused on the 2025 agenda and its implementation
- Strategic partnerships: collaborating with our doctors, government, research providers and other organisations
- A continuous improvement culture: fostering an environment that is adaptive to change and quality improvement

Supplementary File S2. Delphi Survey

This survey is the first of two parts in a study that aims to capture your insights, beliefs and experiences to inform priorities for implementing gender equity interventions.

Gender equity interventions included in this survey are extracted from two sources:

1. A recent systematic review of cross sector evidence on organisational activities that **advance women in leadership.**

2. Interview data with women from **Epworth's leadership** who have experienced the career advancing impact of interventions (from within and outside of Epworth).

In this Initial survey, we are interested in what you **believe to be important** and **acceptable** from a full suite of interventions.

This survey should take no longer than 10 minutes.

<u>Part 1 of the Delphi: Initial priority setting survey at Epworth</u>

Section 1.

Select <u>ALL</u> interventions you believe are most important for delivering gender equity outcomes that advance women into leadership at Epworth.

- Committed and supportive leadership team
- Internal communication strategy of Epworth's commitment towards gender equity
- Formal mentoring program for women
- Informal mentorship for women
- Leadership training and development for women
- Nominating employees to attend leadership training
- Career development opportunities or online learning
- Flexibility in work hours i.e. job-sharing opportunities
- Flexibility in work schedule i.e. autonomy in location/ mode of work (remote, online)
- Flexibility with work location: ability to work from home when needed
- Flexibility of work practices reflected in the Enterprise Bargaining Agreement (EBA)
- Recruitment policies targeting diversity of workforce
- Recruitment policies targeting diversity of leadership
- Provision of scholarships for career development
- Transparency around award rates in EBA
- Transparency around promotion processes
- Strategy for gender pay equality
- Supportive and positive organisational culture
- Encouraging women to self-nominate for leadership positions
- Shoulder tapping for leadership opportunities
- Succession planning and feeder processes
- Parental /carer leave arrangements for both men and women
- Formal succession planning and role feeder processes
- Gender equity targets i.e. aspirational goals
- Gender equity quotas i.e. mandatory outcomes
- Increased support for VMOs to realise career aspirations

- Formal celebration and acknowledgement of women’s achievements
- Celebration and acknowledgement of women’s achievements
- On-site childcare services for parents
- On-site breast milk expressing spaces
- Training module on gender bias
- Opportunities and resources for coaching and well-being
- Building and maintaining employee resource groups for women i.e. women-only networking
- Mixed networking events for women
- Explicit internal communication of Epworth's commitment towards gender equity

*Multiple selections enabled/ Forced response.

Section 2.

The following statements are related to your beliefs around what is important for an organisation like Epworth to do (future focused and action-based) in order to advance women in leadership.

Rating scale for 1-8 (single selection/forced response).

Extremely important
Very important
Somewhat important
Not so important
Not at all important

1. The organisation and its leaders need to explicitly support and encourage women to **apply** for leadership roles
2. The organisation needs to actively **promote** the visibility of women in positions of leadership. i.e. present them as role models
3. The organisation needs to provide **flexible work options** to enable **access** to equal opportunity
4. The organisation needs to create **opportunities** for women to progress and advance
5. The organisation needs to provide women with **tools and resources** to actively manage and advance their careers
6. The organisation needs to equip its leaders with **new skills** such as emotional intelligence, empathy and compassion to effectively manage performance in a flexible/changing model
7. The organisation needs to provide **sponsorship** for women where a senior employee acts on behalf of the sponsored, actively advocating for them and opening up opportunities for advancement and promotion
8. The organisation needs to provide **education and training** on gender issues and the challenges women face at work

---

Section 3.

The following questions are focused on the implementation of gender equity interventions, with an interest in your current perceptions of Epworth. (1-4= single selection/forced response).

1. How would you rate Epworth's current **capability** to implement gender equity interventions?

Extremely capable

Very capable

Somewhat capable

Not so capable

Not at all capable

Explain: [Box] open response

2. How would you rate Epworth's **readiness** to implement gender equity interventions?

Extremely ready

Very ready

Somewhat ready

Not so ready

Not at all ready

Explain: [Box] open response

3. What level of **support** do staff need from your management/leadership team to accept gender equity interventions?

A great deal

A lot

A moderate amount

A little

None at all

Explain: [Box] open response

4. What level of **knowledge** to you believe your **operational leadership** have in relation to **implementing** gender equity interventions?

A great deal

A lot

A moderate amount

A little

None at all

Explain: [Box] open response

Section 4.

The following questions are focused on the support your organisation may need for implementing gender equity interventions

5. What additional support would you like to assist you in implementing gender equity interventions? *Select **ALL** items you believe are **important** for implementing gender equity for women at Epworth*

More Case Studies - examples of what was implemented previously

More Case Studies - examples of HOW to implement gender equity interventions

More specific examples for my sector

More webinars sharing the process of how to implement gender equity interventions
Small group peer support to share safely how the work is progressing
Training - short sharp (1 to 2 hour) sessions on specific steps in the process
Training - 1 to 2 days to go into detail about the process
Support for leadership and management in organisations to understand how to support this work
Demonstration of how to include a gender equity intervention into a program, policy or service
Demonstration of how to include a gender equity intervention into a major/large strategy (whole department, region, state)
Guidance and tips on how gender equity interventions can be implemented across the organisation and become "business as usual"
Tools to support implementation

*Multiple selections enabled/ Forced response.

6. Are there any further resources, training or support that would assist you and your organisation?

[Box] open response

7. Are there additional barriers or challenges that you or your organisation might experience when implementing gender equity interventions?

[Box] open response

Thanks for participating in this survey!

You will receive a second survey shortly. This will be a ranking exercise, and will contain questions based on your responses here today. The second survey will take no longer than 5 minutes and needs to be completed within the time allocated.

We value your time and really appreciate the insights and input from your experience. Your answers will assist us in understanding the support and resources that you may need to strengthen the implementation of gender equity interventions within your organisation.

For any questions, feedback or comments, please contact:

mariam.mousa@monash.edu

End.
